# Rheology and Culture Reproducibility of Filamentous Microorganisms: Impact of Flow Behavior and Oxygen Transfer During Salt‐Enhanced Cultivation of the Actinomycete *Actinomadura namibiensis*

**DOI:** 10.1002/elsc.202400078

**Published:** 2024-11-27

**Authors:** René Hanke, Jonas Lohr, Leon Poduschnick, Sebastian Tesche, Luc Fillaudeau, Jochen Büchs, Rainer Krull

**Affiliations:** ^1^ AVT.BioVT – Chair of Biochemical Engineering Rheinisch‐Westfälische Technische Hochschule (RWTH) Aachen University Aachen Germany; ^2^ Institute of Biochemical Engineering Technische Universität Braunschweig Braunschweig Germany; ^3^ Center of Pharmaceutical Engineering (PVZ) Technische Universität Braunschweig Braunschweig Germany; ^4^ Toulouse Biotechnology Institute (TBI) Université de Toulouse, CNRS, INRAE, INSA Toulouse France

**Keywords:** filamentous bacteria, online monitoring, Respiratory Activity MOnitoring System (RAMOS), rheology, shake flasks

## Abstract

Analyzing the relationship between cell morphology, rheological characteristics, and production dynamics of cultivations with filamentous microorganisms is a challenging task. The complex interdependencies and the commonly low reproducibility of heterogeneous cultivations hinder the bioprocess development of commercially relevant production systems. The present study aims to characterize process parameters in *Actinomadura namibiensis* shake flask cultures to gain insights into relationships between culture behavior and rheological characteristics during salt‐enhanced labyrinthopeptin A1 production. Plate–plate (PP) and vane–cup rheometer measurements of viscous model fluids and culture broths are compared, revealing a more uniform distribution of broth when measured with the PP system. Additionally, rheological characteristics and culture performance of *A. namibiensis* cultures are evaluated using online data of the specific power input and the oxygen transfer rate. It is demonstrated that salt‐enhancement labyrinthopeptin A1 production by the addition of 50 mM (NH_4_)_2_SO_4_ increases the apparent viscosity of the *A. namibiensis* culture by four‐fold and significantly reduces the reproducibility of the culture resulting in a 46 h difference in lag‐phase duration. This approach demonstrates that the culture behavior of complex filamentous cell morphologies is challenging to decipher, but online monitoring of rheology and oxygen transfer can provide valuable insights into the cultivation dynamics of filamentous microbial cultures.

**Abbreviations**
CDW: cell dry weight
DOT: dissolved oxygen tension
intOTR: integrated oxygen transfer rate
OTR: oxygen transfer rate
PP: plate–plate
RAMOS: Respiratory Activity MOnitoring System
VC: vane–cup

## 1 Introduction

Actinomycetes are valuable sources for the production of biopharmaceuticals [1]. However, the availability of products derived from actinomycetes is generally low, as a deeper understanding of regulation mechanisms and systematic approaches for their targeted cultivation processes is still lacking [2]. Actinomycetes grow filamentously showing characteristics similar to those of fungi. Starting from germinating spores, single hyphae continue to grow at their tips or branches and form a three‐dimensional mycelial network [3, 4]. Depending on the cultivation conditions, freely dispersed hyphae may aggregate to form loose clumps or dense pellets. In many cases, a clear relation between culture morphology and productivity can be observed [5, 6, 7, 8], which makes further bioprocess development to a complex interplay between productivity, culture rheology, and culture morphology. Although a correlation between labyrinthopeptin A1 production and culture morphology has been reported for *A. namibiensis* [9], the culture rheology of *A. namibiensis* has not been linked to labyrinthopeptin A1 production or culture morphology.

**Summary**

- Understanding, monitoring and characterizing the interactions of cell morphology, broth viscosity and culture productivity remain a prominent challenge in the cultivation of industrially relevant filamentous organisms, which often display shear rate-dependent flow behavior and a low culture reproducibility.
- The comparison between different rheometer systems using suitable viscous model fluids and *Actinomadura namibiensis* culture broth provides a guide to choose a reliable measurement instrument for the characterization of filamentous bacteria with complex morphology, for the comparison of rheological data and to facilitate technology transfer.
- Comparative analyses of salt-enhanced and non-supplemented labyrinthopeptin A1 production in *A. namibiensis* shake flask cultures focused on the rheological properties and reproducibility of the cultivation phases based on sampled data and online monitoring of apparent viscosity and oxygen transfer rate.
- The deeper insights into the reproducibility of *A. namibiensis* cultures can serve as blueprint for the characterization of other filamentous organisms with complex morphology.

The complex morphology of filamentous organisms can influence biotechnological processes in many ways. Depending on the predominant morphology in the culture broth, the rheology of the culture broth changes, which in turn affects heat and mass transfer as well as mixing, among others [10]. Although the exact relationship between cellular morphology and broth viscosity is challenging and depends on the organism used, it has been shown that rheological parameters can be described by morphological descriptors, like the morphology number or the lacunarity in the cultivations of *Aspergillus niger* [8, 11]. From a macromorphological point of view, pellet‐like morphologies usually lead to a lower viscosity of the culture broth and easier downstream processing; however, they can lead to transfer limitations within the pellet core. Freely dispersed mycelia, on the other hand, have a higher viscosity of the broth but can promote growth and product formation [12]. Certain morphologies are often preferable to others if a specific product is to be synthesized [13, 14]. To ensure optimal production conditions, it is therefore crucial to assess the morphological development as well as the rheology of the culture broth in the course of a cultivation. Therefore, many efforts have been made to effectively determine and control the morphology or rheology of filamentous cultures [11, 15–17].

In general, rheometers can be divided into two classes, which are characterized by either an absolute or a relative measurement of the sample viscosity [18]. Absolute measurement systems, such as rotational rheometers, are the gold standard for rheological characterization. However, they require samples to be taken from the culture broth and are, by definition, dependent on certain geometries of the measurement setup. Therefore, online in‐situ monitoring of the viscosity of the culture broth is not possible with this type of device. In addition, differences in the geometry of the measuring systems between the manufacturers make it difficult to transfer knowledge between the laboratories carrying out the measurements. This is especially true for vane–cup (VC)‐type rheometers, as their applicable shear rate range is limited at the lower end by the sedimentation of cells/pellets and at the upper end restricted by the transition to a turbulent flow regime [19]. Since both limits depend on the VC geometry and the broth rheology of the organism under investigation, direct comparisons of rheological parameters should be treated with caution. However, the in‐situ implementation of relative measurement systems is possible. One approach is to measure the torque generated by the movement of the broth on the shaker axis at a certain shaking frequency during shake flask cultivations, which was demonstrated to be a feasible method for the filamentous bacterium *Streptomyces lividans* [20]. In this way, the apparent viscosity *η*_app_ of the culture broth can be calculated under *in‐phase* conditions (for more details see Supporting Information) [21, 22].

Sieben et al. [23], Halmschlag et al. [24, 25], and Hoffmann et al. [26] demonstrated an alternative approach that monitors *η*_app_ of a culture broth based on the angular shift of the leading edge of the bulk liquid in a rotating shake flask. The measurement principle is based on the change in liquid distribution caused by the increased friction between the liquid boundary and the shake flask wall as viscosity increases. By monitoring the trend of *η*_app_ online over the course of a cultivation, morphological changes and thus changes in productivity can be identified.

Although the course of *η*_app_ is crucial for the quantification of rheological characteristics, it provides only limited information about the growth dynamics, substrate consumption, or the metabolic activity of microbial cultures. However, monitoring the oxygen transfer rate (OTR) using the Respiratory Activity MOnitoring System (RAMOS) helps to investigate the physiology of aerobic microorganisms, as important metabolic activities depend on oxygen consumption [27, 28, 29, 30, 31]. Similarly, salt‐enhanced production of labyrinthopeptin A1 by *A. namibiensis* has been reported to be linked with increased oxygen consumption compared to nonsupplemented conditions [32]. The benefits of monitoring both, *η*_app_ and the OTR, have already been demonstrated for biopolymer production [33, 34, 35]. Giese et al. [36] applied this dual monitoring to investigate the growth of the filamentous fungus *Trichoderma reesei*. To the best of our knowledge, dual online monitoring of specific power input and OTR of a filamentous bacterial culture in shake flasks has not been published so far.

This study aims to provide support in the selection of a suitable rheometer setup for the characterization of a filamentous bacterial culture. This is demonstrated by comparing rheological measurements of viscous model fluids as well as *A. namibiensis* culture broth using a plate–plate (PP) and a VC system. Furthermore, the dual online monitoring of *η*_app_ and OTR is demonstrated to indicate morphological, rheological, and physiological changes of an *A. namibiensis* culture in shake flasks. Finally, a simple method to characterize the reproducibility of individual culture phases by time shifting is proposed.

## 2 Materials and Methods

### 2.1 Strain Maintenance, Media, and Cultivation Conditions

The strain *Actinomadura namibiensis* (DSM 6313) used in this study was obtained from the German Collection of Microorganisms and Cell Cultures, Braunschweig, Germany. Agar fragments with a size of 1 cm^2^ of dense cultures stored in 80% glycerol at −80°C were used as starting material. Both precultures and main cultures were prepared using a modified M5294 medium as described previously [32]. Precultures were kept in darkness at 30°C and shaken at 180 min^−1^ for 3 days using 70 mL working volume in 500 mL unbaffled shake flasks. Salt‐enhanced main culture medium was supplemented with an additional 50 mM (NH_4_)_2_SO_4_. Inoculation of main cultures was performed using a 5 mL preculture. Main cultures were kept in darkness at 30°C and shaken at 180 min^−1^ for 10 days using 100 mL working volume in 500 mL unbaffled shake flasks [32].

The respiratory activity of *A. namibiensis* cultures was determined via a self‐made respiration activity monitoring system (RAMOS) device [27, 28]. Commercial versions are available (HiTec Zang GmbH, Herzogenrath, Germany). One data point of the OTR was obtained per hour of cultivation. The principle of the measuring device is given in the Supporting Information File (Figure S1).

### 2.2 Offline Analytics of Bioprocess Samples

Cell dry weight (CDW) was determined gravimetrically from 15 mL culture broth [32]. Glucose and glycerol were quantified through analytical HPLC measurements of cell‐free supernatant using a MetaCarb 87C column (300 × 7.8 mm, Agilent Technologies, Santa Clara, USA) [32]. Labyrinthopeptin A1 concentrations were determined from crude culture extracts by a previously established analytical HPLC method using a XBridge Shield RP18 column (5 µm, 250 × 4.6 mm, Waters, Milford, USA) [32, 37].

### 2.3 Specific Power Input Measurement

Online measurements of the specific power input of *A. namibiensis* shake flask cultures were performed in a custom‐made device [21, 22]. This device measures the torque at the shaker axis at a given shaking frequency. The tableau of the shaker is loaded with nine 500 mL Erlenmeyer flasks, the liquid content of which contributes in total to the measured torque at the shaker axis. Measurements of torque and shaking frequency were taken at a sample rate of 5 s^−1^. The gathered values were averaged over an interval of 12 min (3600 data points) throughout the experiment. *η*_app_ of the culture broth was determined from the torque measurements, as described previously elsewhere [21, 22]. A detailed description of the applied algorithm is given in the Supporting Information.

### 2.4 Rheological Measurements

Rheological measurements were carried out at two different locations with equipment from two different manufacturers. Initially, viscous model fluids were used to compare the rheometers used in this study. The equipment used is summarized in Table 1. As viscous model fluids, 0.15% and 0.5% (w/w) xanthan solutions (Food grade E415, Fufeng, Shadong RPC) with shear‐thinning behavior were chosen, as they mimic the rheological behavior of *A. namibiensis* culture broth [19]. To prevent microbial spoilage of the solutions, 0.1% (w/w) sodium azide was added.

**TABLE 1:** Geometries of rheometer systems at two different laboratories.

Location	Rheometer	Measuring system	Diameter (mm)		Gap size (mm)	Impeller height (mm)
Aachen	Anton Paar Physica MCR 301	Plate–plate (PP)	Upper plate	49.95	1.5	—
			Lower plate	55		
Aachen	Anton Paar Physica MCR 301	Vane–cup (VC)	Vane cup	26.0	—	40
				28.9		
Braunschweig	Malvern Kinexus Lab+	Plate–plate (PP)	Upper plate	60	1.5	—
			Lower plate	65		
Braunschweig	Malvern Kinexus Lab+	Vane–cup (VC)	Vane cup	25.0	—	61
				27.5		

After loading the respective measuring systems with viscous model fluid or culture broth, the sample was covered with a hood and was tempered to 30°C for 5 min. Then, a sequence of shear rates $\dot{\gamma }$ between 0.1 and 500 s^−1^ with six logarithmic steps per decade was applied. Each shear rate was held until the instrument's steady‐state criterion was met. In the PP systems, the test was performed from low to high shear rates and back to low shear rates, to check for time‐dependent flow behavior. In the VC system, the test was performed from high to low shear rates and back to high shear rates, as the biomass settled in the tempering step.

### 2.5 Determination of Rheological Parameters

Rheological parameters were estimated by method of least squares from individual shear rate tests. The Herschel–Bulkley model was chosen to describe the observed shear‐thinning behavior [38] (Equation 1), as preliminary experiments showed that *A. namibiensis* shake flask cultures exhibited a static yield stress (*τ*_0_) greater than 0 mPa s (see Table S1). The model describes the relationship between the shear stress τ, the yield stress $\tau_{0}$, the flow consistency factor K, the shear rate $\dot{\gamma }$, and the flow behavior index m.

(1) $$\tau =\tau_{0}+K\cdot {\dot{\gamma }}^{m}$$

For each individual shear rate test, the interval of shear rates, to which a fit was applied, was determined by finding the interval, where the gradient of *η*_app_ was constant with statistically significance (*F*‐test, α<0.05) over the logarithmically plotted $\dot{\gamma }$. Curve fitting was done with the “curve_fit” function of the SciPy Optimize package in Python 3.7.3 [39]. The Reynolds number (*Re*) for the VC system was calculated according to Equation (2), where *ρ* is the liquid density of 1000 kg m^−3^, *n* is the stirring frequency in s^−1^, *D* is the diameter of the vane instrument in m (Table 1), and *η*_app_ is the apparent viscosity in Pa s.

(2) $$Re=\frac{\rho \cdot n\cdot D^{2}}{\eta_{\mathrm{app}}}$$

## 3 Results and Discussion

### 3.1 Comparison of Rheometers Using Model Fluids

**FIGURE 1:**
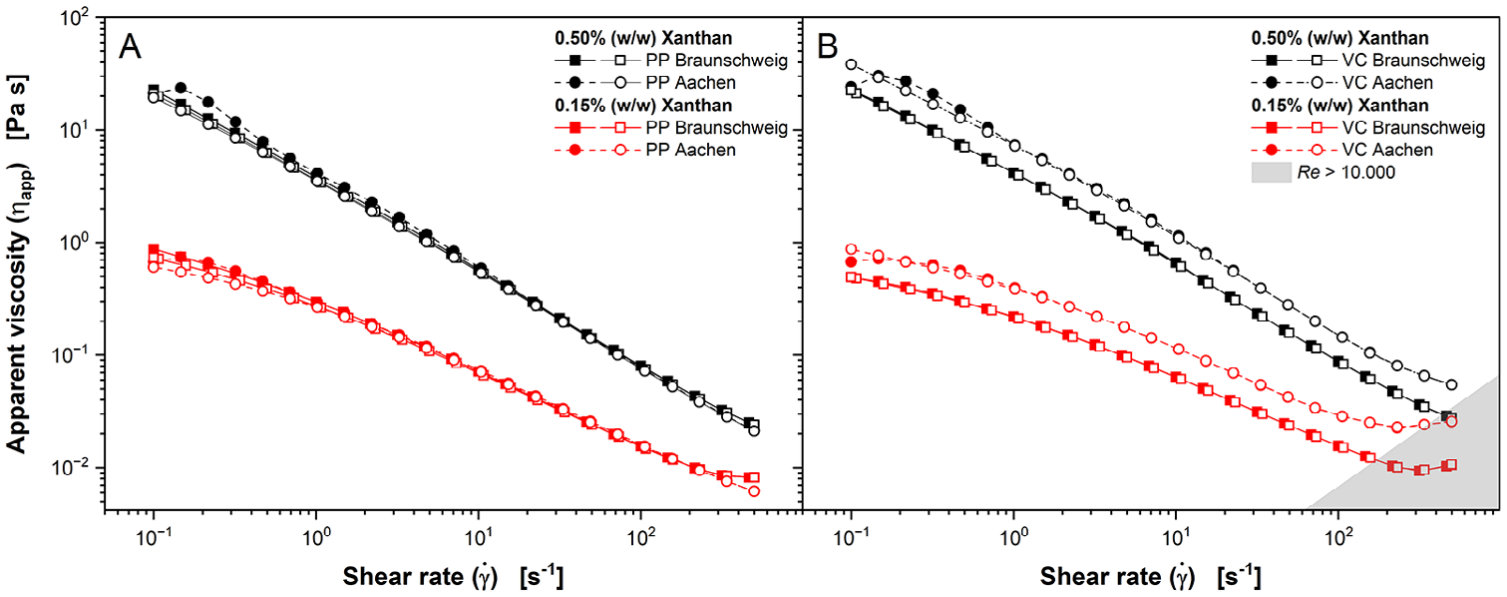
Apparent viscosity *η*_app_ of xanthan solutions measured at two different locations using (A) a plate–plate (PP) and (B) a vane–cup (VC) system. Measurements at RWTH Aachen University performed using instruments by Anton Paar GmbH, measurements at TU Braunschweig performed using instruments by Malvern Panalytical Ltd. Measurements were performed at a temperature of 30°C with six logarithmic steps per decade in steps from low to high shear rates (filled symbols) and back to low shear rates (open symbols) for the PP system and in steps from high to low shear rates (open symbols) and back to high shear rates (filled symbols) for the VC system. Therefore, every condition was measured twice. The Reynolds number (*Re*) for the VC system was calculated according to Equation (2).

The rheometers used in this study were compared by measuring xanthan solutions of two concentrations (0.15% and 0.50% [w/w], Figure 1). As expected, the xanthan solutions showed a shear‐thinning behavior for the PP and the VC measuring system. The measured apparent viscosities are within the reported range for *A. namibiensis* culture broth [19]. For the PP system (Figure 1A), $\dot{\gamma }$ ramps from low to high $\;\dot{\gamma }$ and back to low $\dot{\gamma }$ deliver equal results. Slight offsets are visible at very low $\dot{\gamma }$ as the torque measurements of the rheometers are most prone to error at these conditions [40]. For the 0.15% (w/w) xanthan solution and high $\dot{\gamma }$, the viscosity curve starts to plateau. This indicates a turbulent flow regime forming in the measurement device. Contrary, this was not observed for the 0.5% (w/w) solution, due to its higher viscosity. Interestingly, for the VC device (Figure 1B), there is an offset between the measurements performed using the two different rheometers. Again, $\dot{\gamma }$ ramps, illustrated by filled and open symbols, deliver equal results. Beginning turbulence, illustrated in Figure 1B as a gray shaded area with *Re* > 10.000, can be detected at higher $\dot{\gamma }$ of 10^2^ s^−1^ for the Anton Paar rheometer (Aachen) due to the different geometry of the VC tool used (Table 1).

Overall, the measurements show that PP devices provide a more reliable comparison of rheological characteristics. However, samples containing biopellets may influence the measurement [41, 42]. For measuring *A. namibiensis* culture broth, this is irrelevant, as the majority of cell pellets is smaller than 200 µm [32]. As a rule of thumb, the gap between the two plates should be chosen 10 times the pellet diameter to avoid pellet influence [42]. Based on those results, the PP devices were chosen to measure *A. namibiensis* culture broth in this study.

### 3.2 Practical Insights of Measuring *A. namibiensis* Culture Broth: Rotational Rheometer Tests With Different Measuring Devices

**FIGURE 2:**
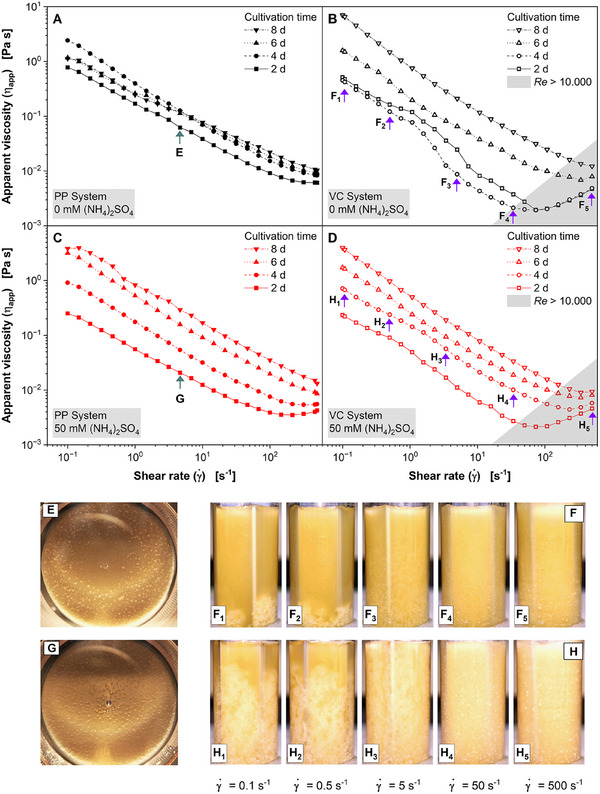
Apparent viscosity *η*_app_ of the cultivation broth of *A. namibiensis* and distribution of biomass in a self‐made transparent acrylic glass rheometer. *A. namibiensis* cultivated at *V*_F_ = 500 mL, *V*_L_ = 100 mL, *T* = 30°C, *n* = 180 min^−1^, *d*_0_ = 50 mm. (A, B, E, F) Without and (C, D, G, H) with 50 mM (NH_4_)_2_SO_4_‐supplementation of the culture medium. (A and C) *η*_app_ as function of shear rate, determined with the plate–plate (PP) system measured in steps from low to high shear rates. Green arrows indicate conditions of photos taken by a self‐made transparent acrylic glass PP rheometer in (E and G). (E and G) Samples taken after 2 days of main cultivation and measured using the self‐made transparent acrylic glass PP rheometer system at $\dot{\gamma }\;=\;5$ s^−1^. (B and D) *η*_app_ as a function of shear rate, determined with the vane–cup (VC) system measured in steps from high to low shear rates. Violet arrows indicate conditions of photos of self‐made transparent acrylic glass VC rheometer in (F and H). (F and H) Samples taken after 4 days of main cultivation and measured using the self‐made transparent acrylic glass VC rheometer during a shear rate test from $\dot{\gamma }\;=\;0.1$ s^−1^ to $\dot{\gamma }\;=\;500$ s^−1^. The Reynolds number (*Re*) for the VC system was calculated according to Equation (2).

To evaluate which measuring device yields more reproducible results for the rheological characterization of *A. namibiensis*, shear rate tests of culture broths were carried out with both measuring geometries. In the PP system, the test was always performed with gradients from low to high $\dot{\gamma }$ (filled symbols), to avoid possible destruction of the hyphal network. In the VC system, the test was performed from high to low $\dot{\gamma }$ (open symbols) to minimize the influence of biomass sedimentation. In Figure 2A–D, *η*_app_ of the cultivation broth at different cultivation times is displayed as a function of $\dot{\gamma }$ for the nonsupplemented culture and the culture with 50 mM (NH_4_)_2_SO_4_ addition. All flow curves decline by up to two orders of magnitude, when increasing $\dot{\gamma }$ from 0.1 to 100 s^−1^. This indicates a strong shear‐thinning flow behavior. In the PP system, 𝜂_app_ of the culture without addition of (NH_4_)_2_SO_4_ rises with increasing cultivation time for $\dot{\gamma }$ > 10 s^−1^, but at lower $\dot{\gamma }$ the trend is not clear (Figure 2A). The salt‐enhanced cultivation broths with addition of (NH_4_)_2_SO_4_ show a continuous and stronger increase of 𝜂_app_ over time in the tested range of $\dot{\gamma }$ (Figure 2C) with lower values of 𝜂_app_ at Day 2 and higher values of 𝜂_app_ at Days 6 and 8 of cultivation, compared to the viscosity values of the nonsupplemented culture. The lower initial *η*_app_ in case of salt‐enhanced cultivation can be explained by the slower growth of this culture [32], whereas the stronger increase may be attributed to the differences in the cellular morphology observed between both cultivation conditions [9]. The result of the measurement with the VC system is quite different. In the nonsupplemented culture (Figure 2B), the flow curves have a greater distance to each other, but the curves obtained for Days 2 and 4 are very uneven, indicating large measurement uncertainties. These uncertainties remained when measurements of samples were repeated, and they were also observed in low‐to‐high shear rate tests (data not shown). The results from the salt‐enhanced cultivation samples measured in the VC system (Figure 2D) were similar to those measured in the PP system (Figure 2B). However, in the VC system 𝜂_app_ considerably increased at $\dot{\gamma }$ > 100 s^−1^, especially at Day 2 of cultivation. This is likely caused by turbulent flow or the occurrence of Taylor vortices at high $\dot{\gamma }$, which more easily occurs in low‐viscosity samples [43].

The tighter mycelial network of the salt‐enhanced cultivation is potentially more rigid, which could explain the strongly increasing *η*_app_ from Days 4 to 10 of this cultivation and the comparatively small increase of 𝜂_app_ of the nonsupplemented cultivation in this timeframe [9]. However, a higher flexibility of hyphae is expected to lower the viscosity of filamentous cultivation broths due to lower resistance of the hyphae against alignment in the direction of flow. The salt‐enhanced cultivation has a lower 𝜂_app_ than the nonsupplemented culture only at the beginning of cultivation. Based on these results, it can be assumed that disentanglement and alignment of hyphae upon shear is only possible at low biomass concentrations as long as the hyphae are not yet strongly intertwined [9].

The results depicted in Figure 2A–D do not clearly show, whether the results of the PP system or the VC are more reliable. Thus, replicas of transparent acrylic glass (PMMA) were made for both systems, enabling the visual observation of the samples during the test sequence. Throughout the measurement in the $\dot{\gamma }$ range from 0.1 to 500 s^−1^, the biomass in the 1.5 mm gap of the PP system was distributed evenly. Figure 2E, G shows an example of the biomass distribution at $\dot{\gamma }$ = 5 s^−1^. Small air bubbles that sometimes occurred in the middle of the base plate were considered not to affect the measurement, because $\dot{\gamma }$ in the PP system is radially dependent and should be negligibly small in the center of rotation. Since stainless steel and PMMA have different surface properties, the adhesion in the replica system may have differed from the original PP system. However, the obtained values of 𝜂_app_ were very similar for both PP systems. Therefore, it is assumed that the distribution of the biomass is equal in the original and the replicated PP system.

The observation of the measurement in the transparent replica VC system (Figure 2F, H) revealed a major drawback of the vane geometry: with a decreasing $\dot{\gamma }$ the measurement time increased and the pellets and free mycelia of the nonsupplemented culture settled rapidly (Figure 2F_1‐2_). At $\dot{\gamma }$ = 5 s^−1^, the biomass was already mainly located in the lower half of the cylinder. At $\dot{\gamma }$ between 0.1 and 0.5 s^−1^, the biomass was completely sedimented and moved in the direction of rotation by the vane. Such a sediment layer in the measuring cylinder is often called *cake* [44]. At low $\dot{\gamma }$, the cake acts like a brake on the rotor. This state is reflected in a steep upward flow curve and may be the reason for the unexpected results in Figure 2B. A similar effect of sedimentation was also observed with the salt‐enhanced cultivation broth (Figure 2H), but in this case the sedimentation velocity was slower. The mycelial network of the salt‐enhanced cultivation broth appeared to be more rigid, which is in accordance to its more rapidly increasing viscosity over the course of the cultivation time (Figure 2C, D). This also suggests that the degree of entanglement of hyphae is higher in the salt‐enhanced cultivation broth [9].

### 3.3 Salt‐Supplementation Increases *A. namibiensis* Culture Broth Viscosity

Rheological parameters of *A. namibiensis* culture broth were determined from shear rate tests conducted from daily samples of salt‐enhanced ($50\;\mathrm{mM}\;{\left(NH_{4}\right)}_{2}SO_{4}$) and control cultivations ($0\;\mathrm{mM}\;{\left(NH_{4}\right)}_{2}SO_{4}$). The *η*_app_ was determined in separate cultivations performing measurements of the specific power input. For cultivations of filamentous microorganisms with complex morphology, rheological parameters of the culture broth may reveal information on the current predominant morphology and, thus, can serve as a mean for process control and optimization [11, 45].

**FIGURE 3:**
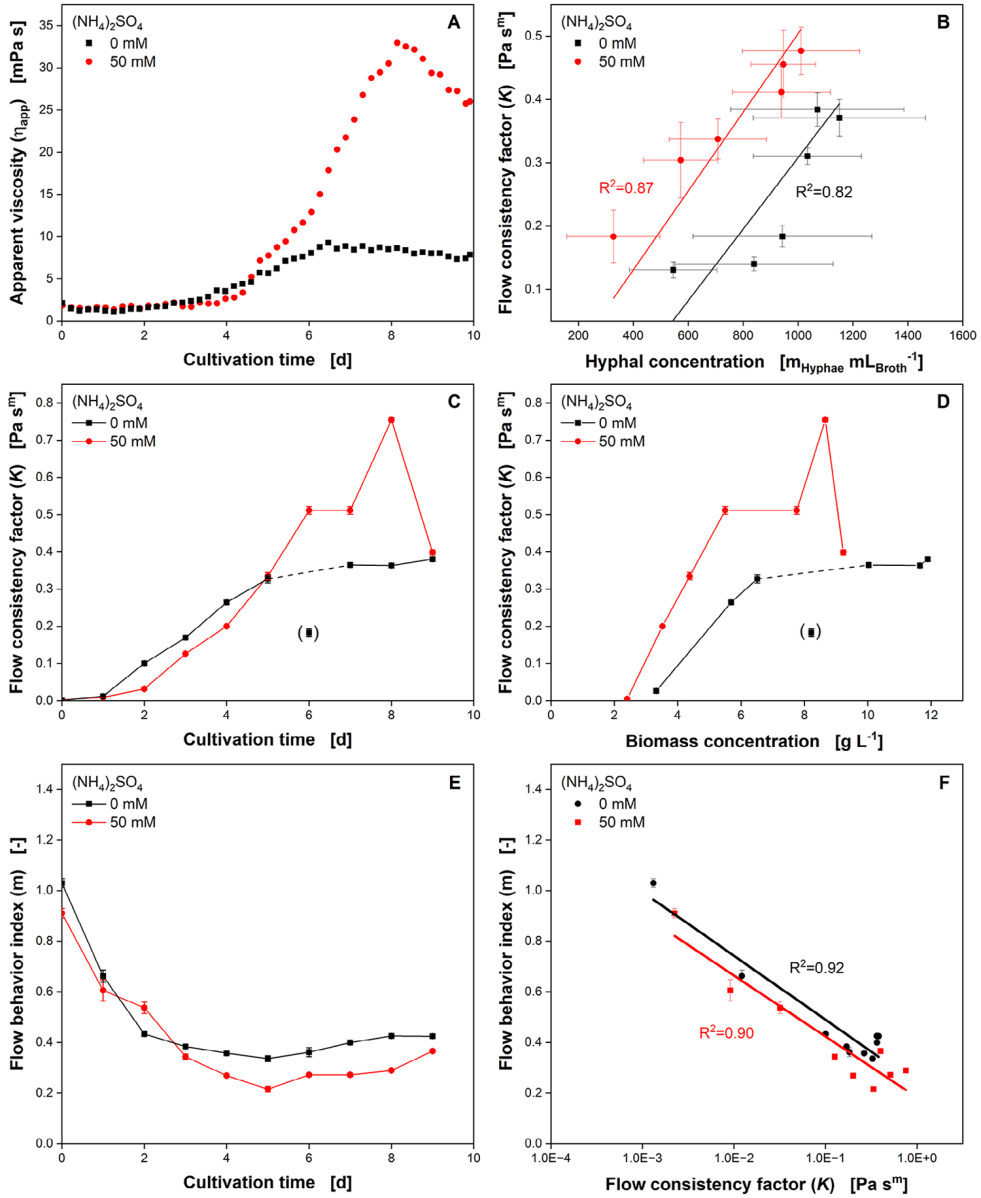
Rheological and biological conditions of *A. namibiensis* cultures with and without 50 mM (NH_4_)_2_SO_4_‐supplementation of the culture medium: (A) apparent viscosity *η*_app_ is calculated based on power input measurements, according to Büchs et al. (2000), (B) correlation between flow consistency factor *K* and the hyphal concentration, (C) flow consistency factor *K* over cultivation time, (D) correlation between flow consistency factor *K* and the biomass concentration, and (E) flow behavior index *m* over cultivation time. Error bars indicate the standard deviation for *m*, obtained by the Herschel–Bulkley fit, (F) correlation between flow behavior index *m* and flow consistency factor *K*, according to Henzler et al. (1987). *V*_F_ = 500 mL, *V*_L_ = 100 mL, *T* = 30°C, *n* = 180 min^−1^, *d*_0_ = 50 mm. (C, D) data in brackets is assumed to be an outlier.

Figure 3A shows *η*_app_ of *A. namibiensis* shake flask cultivations, while Figure 3C depicts the flow consistency factor K over the cultivation time. *η*_app_ determined with the power input measurements (Section 2.3) starts at approximately 1 mPa s and slowly increases to values of 4 mPa s during the first 4 days, for both, the salt‐enhanced and the control cultivation. Afterwards, *η*_app_ of the control cultivation slightly increases until reaching a plateau at approximately 8.5 mPa s after roughly 6 days, while *η*_app_ of the salt‐enhanced culture increases to values of 33 mPa s after 8 days of cultivation. The observed $\eta_{\mathrm{app}}$ trends are reflected in the respective value *K* (Figure 3C). However, the deviation between the salt‐enhanced and the control culture appears to be less pronounced considering the flow consistency factor K. Although K shows a steep increase to values of approximately 0.3 Pa s*^m^* starting from the second day for both cultivation conditions, the flow behavior index m decreases from values of 1.0 to 0.4 and lower (Figure 3E). The higher *η*_app_ of the salt‐enhanced culture toward the end of the cultivation might be related to the higher pellet fraction of smaller pellets and lower hyphal network spacing [9, 32]. This would also explain the lower biomass and hyphal concentration of the salt‐enhanced cultivation for the same K compared to the nonsupplemented control culture (Figure 3B, D). For both cultivation conditions, *K* correlates linearly with the hyphal concentration, yielding a coefficient of determination of 0.82 and 0.87, respectively (Figure 3B). Changes in pellet fraction and pellet size distribution during cultivation under both culture conditions could also explain why there is no clear correlation between *K* and the biomass concentration or other rheological and morphological characteristics (Figure 3D, Figure S2). The plot of m over K according to Ostwald–de Waele power law can be regarded as a fingerprint of a specific shear‐thinning fluid system [46]. It is demonstrated that both parameters are well correlated also for salt‐enhanced and nonsupplemented cultivations of *A. namibiensis* determined from Herschel–Bulkley fits (Equation 1, Figure 3F). Regression analysis between both culture conditions yields no significant difference between the slopes of the linear equations (*p* = 0.770). However, the cultivation condition has a marginally significant effect on the intercept with the ordinate (flow behavior index *m*; *p* = 0.049). Thus, in general, the salt‐enhanced condition is characterized by a slightly higher degree of shear‐thinning compared to the control condition. This can be attributed to the described differences in morphological development and is further reflected in Figure 3E and is in agreement with the higher degree of entanglement as previously reported [9].

In agreement with the investigation by Bliatsiou et al. [19], the PP rheometer setup provided more reproducible and reliable results than the VC tool. Therefore, the PP setup is recommended for the investigation of the shear rate‐dependent viscosity of filamentous bacteria if their maximum the pellet diameter is not more than one tenth of the PP gap.

### 3.4 Oxygen Transfer Reflects Trends of Biomass Growth and Labyrinthopeptin A1 Production

**FIGURE 4:**
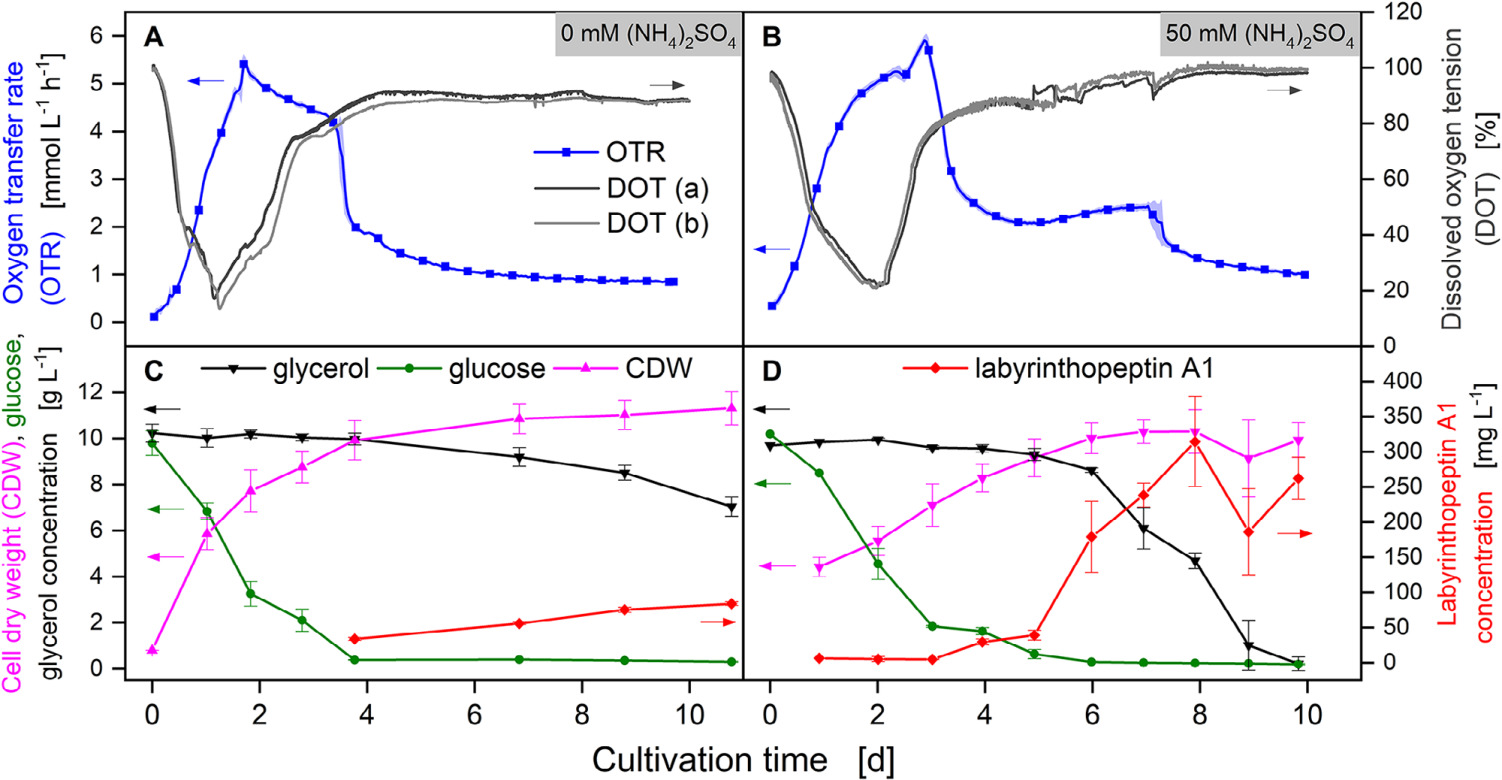
Shake flask cultivations of *A. namibiensis* (A and C) without and (B and D) with supplementation of 50 mM (NH_4_)_2_SO_4_. (A and B) Oxygen transfer rate (OTR) was online monitored using a Respiratory Activity MOnitoring System (RAMOS) device and is given as an average of four biological replicates. For clarity, only four data points per day are shown. The lines are drawn through all data points. Standard deviation is indicated by transparent shadows. Dissolved oxygen tension (DOT) was online monitored with the PreSens shake‐flask reader SFR and is shown as two biological replicates. DOT and OTR were evaluated in different flasks, operated at the same conditions. (C and D) Cell dry weight (CDW), glucose, glycerol, and labyrinthopeptin A1 concentration are shown as average of three biological replicates. Standard deviation is indicated by error bars. Data shown in (C) is modified from Tesche et al. (2019). *V*_F_ = 500 mL, *V*_L_ = 100 mL, *T* = 30°C, *n* = 180 min^−1^, *d*_0_ = 50 mm.

To determine the relevance of oxygen consumption and oxygen transfer for the culture dynamics and the culture characterization, *A. namibiensis* shake flask cultures with and without supplementation of 50 mM (NH_4_)_2_SO_4_ were performed. For both conditions, samples for the determination of glucose, glycerol, CDW, and labyrinthopeptin A1 concentration were taken, and the dissolved oxygen tension (DOT) and the OTR were online monitored (Figure 4).

The initial growth phase of *A. namibiensis* shake flask cultures is characterized by the consumption of glucose and an increase in CDW (Figure 4C, D). Simultaneously, a decrease of DOT and an increase in the OTR can be observed (Figure 4A, B). Although the cultures without supplementation of (NH_4_)_2_SO_4_ show an OTR peak after roughly 2 days (Figure 4A), reaching a CDW concentration of 8 g L^−1^ (Figure 4C), the salt‐enhanced cultures display a later OTR peak after 3 days of cultivation (Figure 4B) and a slightly lower CDW concentration (Figure 4D). The supplementation of 50 mM (NH_4_)_2_SO_4_ influences the morphological development of *A. namibiensis* and affects biomass growth, as indicated by the different courses of the DOT and the OTR for the two culture conditions [32].

It is assumed that the OTR peak at 2 days indicates the time point of the cultivation, when the glucose concentration falls below the threshold, where the product formation coupled with glycerol consumption starts [32]. Concurrently, the beginning of product formation after 4 days can be associated with the observed increase in *η*_app_ (Figure 3A). This observed increase in *η*_app_ in turn can be allocated to the micromorphological development of the culture, which is marked by an increase in hyphal concentration at the onset of the production phase [9].

It is important to note that the data for the OTR trends and the DOT as well as the offline samples come from separate cultivations performed at different laboratories (Aachen and Braunschweig). Although the times of the positive and negative peaks in the OTR and DOT curves do not completely coincide, the general trend is the same for both measurement types. The discrepancy in the signals can be attributed to differences in the individual culture trajectories observed across multiple experiments. The peak shift indicates differences in the lag phase and during glucose metabolism and thus a shift in the onset of the production phase. The onset of glycerol metabolism and thus of the labyrinthopeptin A1 production phase is marked by the formation of an OTR plateau after 4 days (Figure 4A, B). This is the case in both the salt‐enhanced and the control conditions. However, the decrease in glycerol concentration is much steeper in the salt‐enhanced condition, which in turn leads to a higher OTR plateau. A maximum concentration of approximately 314 g L^−1^ labyrinthopeptin A1 is reached after 8 days in the salt‐enhanced cultivation, while the control only reaches a concentration of approximately 65 g L^−1^ after 10.5 days. The differences in labyrinthopeptin A1 concentration can be attributed to the different glycerol consumption (Figure 4C, D). This means that the yield of labyrinthopeptin A1 is relatively constant in both conditions with glycerol, while the yield per biomass and the productivity increase substantially during salt enhancement.

### 3.5 Salt‐Enhancement Increases Labyrinthopeptin Production but Decreases Reproducibility of Growth

**FIGURE 5:**
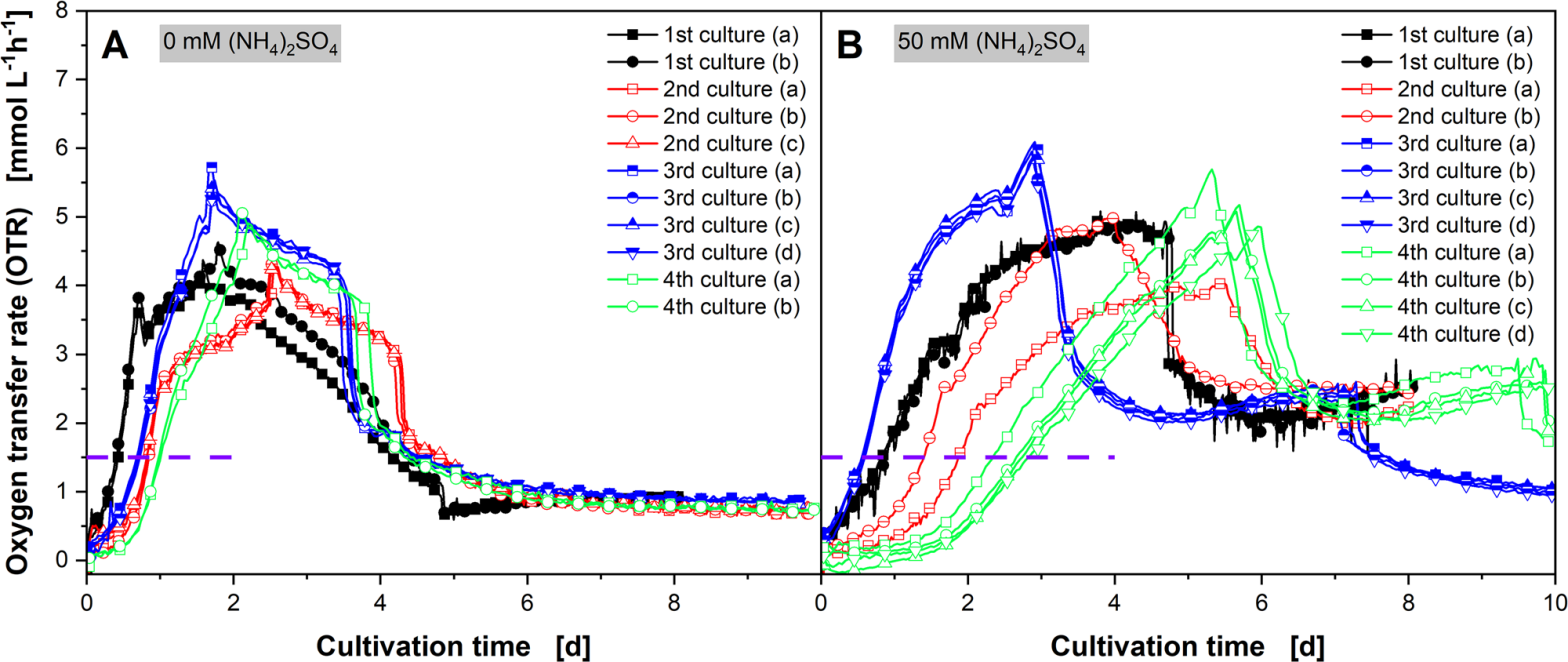
Shake flask cultivations of *A. namibiensis* (A) without and (B) with supplementation of 50 mM (NH_4_)_2_SO_4_ to the culture medium, monitored using a Respiratory Activity MOnitoring System (RAMOS) device. Four cultivations were performed over the course of 3 years with two to four replicates each. The 2nd cultures (a) and (b) are already shown in Tesche et al. (2019). For clarity, only four data points per day are shown. The lines are drawn through all data points. The respective integrated oxygen transfer rates (intOTRs) are shown in Figure S3. *V*_F_ = 500 mL, *V*_L_ = 100 mL, *T* = 30°C, *n* = 180 min^−1^, *d*_0_ = 50 mm. Horizontal dashed purple lines indicate an oxygen transfer rate (OTR) level of 1.5 mmol L^−1^ h^−1^.

Previous works have shown that *A. namibiensis* cultures exhibit heterogeneous in morphology, resulting in a limited reproducibility of the culture behavior [9, 32]. In addition, culture reproducibility can be affected by increased osmotic stress due to salt‐supplementation. To assess the reproducibility of *A. namibiensis* culture behavior and evaluate the effect of salt‐supplementation with 50 mM (NH_4_)_2_SO_4_ on culture reproducibility, the OTR of four independent cultivations was monitored using an RAMOS device (Figure 5). Although OTR trajectories differ between all four experiments, two to four biological replicates within an experiment are in good agreement, given the heterogeneity of *A. namibiensis* cultures. Since the cultivations were carried out over a period of 3 years using the same cryo culture bank was used, this indicates a reliable preculture protocol and highlights the consequences of minor differences in the initial inoculation of the preculture from the cryo culture bank agar fragments (Figure S4).

The time until *A. namibiensis* cultures without salt‐supplementation reached an OTR of 1.5 mmol L^−1^ h^−1^ ranged from 10 h (1st culture) to 1 day (4th culture), showing a difference of up to 14 h in the duration of the lag‐phase (Figure 5A). Initially, all replicates of all four cultures show a steep increase in OTR, indicating similar biomass growth dynamics. The following linear OTR increase, OTR peak, and slow decrease up to 4 days fit well with the biomass growth with a high pellet fraction observed by Tesche et al. [32]. This cultivation phase of *A. namibiensis* with marginal biomass growth is characterized by a reduction of the pellet fraction [32], pellet shrinkage, and an increase of hyphal concentration [9].

The similar OTR trend of *A. namibiensis* cultures shows a reasonable reproducibility under the control conditions without salt addition (Figure 5A). Although the slightly different OTR values indicate different morphological development dynamics during the growth phase, the similar overall integrated oxygen consumption (intOTR) (Figure S3), OTR plateau level, and final labyrinthopeptin A1 titers (Figure S5) indicate a reliable production phase. In contrast, *A. namibiensis* cultures supplemented with 50 mM (NH_4_)_2_SO_4_ showed overall similar OTR trends but large differences of OTR during the growth phase of the replicates for the 2nd and 4th culture (Figure 5B). The time when at which salt‐enhanced cultures reached an OTR of 1.5 mmol L^−1^ h^−1^ ranged from 0.5 days (3rd culture) to 3 days (4th culture), resulting in a difference of up to 2.5 days in the duration of the lag‐phase (Figure 5B). The OTR peaks indicating the end of the growth phase and the beginning of the production phase differ between the different cultures by up to 3 days, with an OTR peak of the 4th culture (day) after 6 days, compared to OTR peaks after approximately 3 days for all four replicates of the 3rd culture (Figure 5B).

**FIGURE 6:**
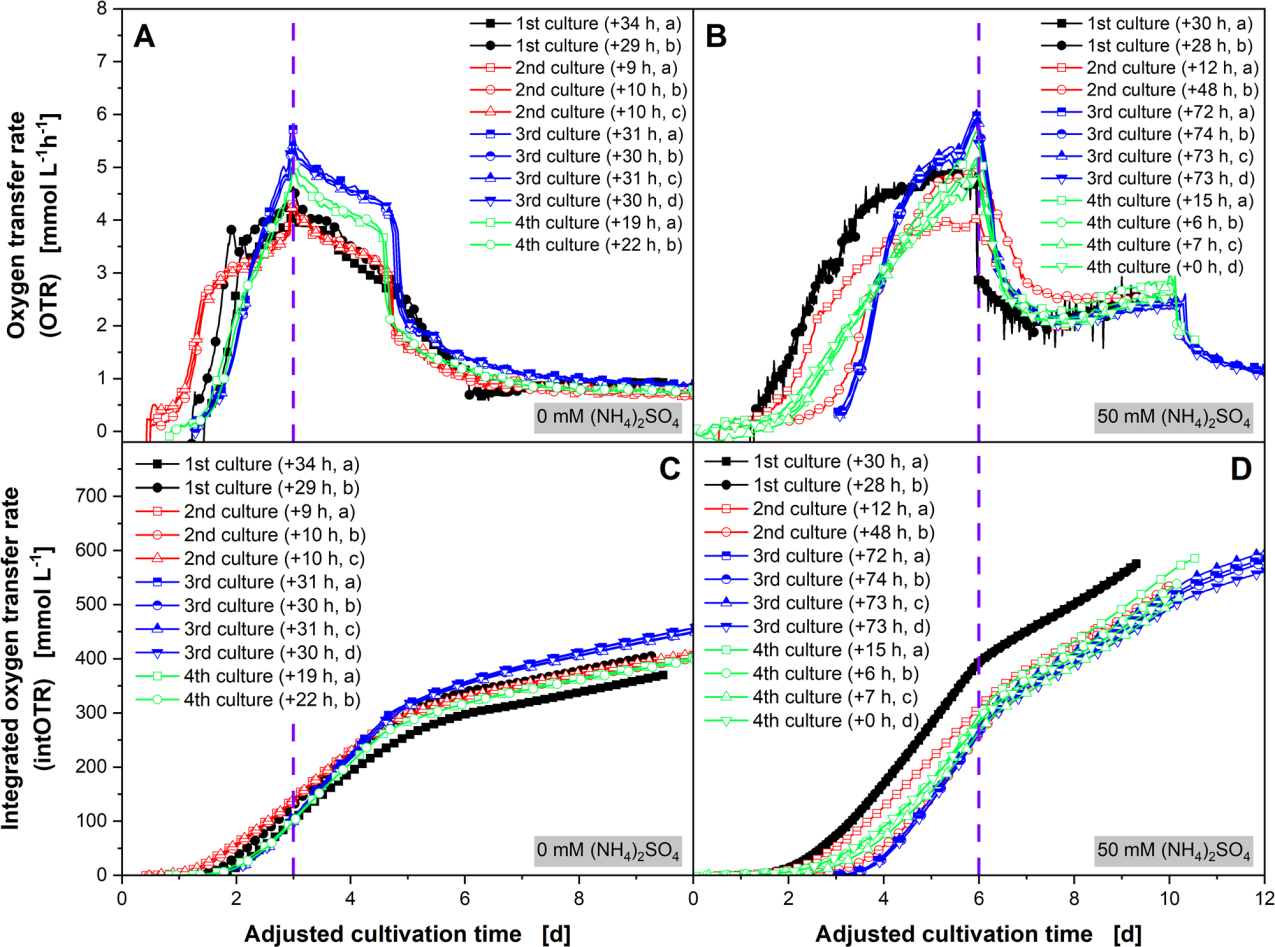
Representation of the oxygen transfer rate (OTR) values (shown in Figure 5) and the integrated oxygen transfer rate (intOTR) values (shown in Figure S3), here temporally shifted by the adjusted cultivation time. (A, C) without and (B, D) with supplementation of 50 mM (NH_4_)_2_SO_4_, monitored with a Respiratory Activity MOnitoring System (RAMOS) device. Four cultivations were performed with two to four replicates each were carried out. The cultivation time to reach the OTR peak is set to *t* = 3 days for nonsupplemented control (A, C) and to *t* = 6 days for salt‐enhanced cultivation (B, D) for each replicate of each individual culture, when the maximum OTR value is reached (indicated by vertical dashed purple line). Time shift to the original cultivation time is indicated in the legends. The 2nd cultures (a) and (b) were taken from Tesche et al. (2019). For clarity, only four data points per day are shown. The lines are drawn through all data points. *V*_F_ = 500 mL, *V*_L_ = 100 mL, *T* = 30°C, *n* = 180 min^−1^, *d*_0_ = 50 mm.

Differences in the duration of the lag phase and the initial growth phase are quite common and are usually tolerated in repeated microbial cultures [25, 47, 48], even when a reliable preculture protocol is followed, and a cryo culture bank is used. However, the inoculation of the preculture using agar fragments might introduce small differences, which may go unnoticed if no online monitoring is performed. If monitored, culture data may be shifted according to a reference point [49, 50]. To eliminate differences in the lag and initial growth phase of the *A. namibiensis* and to focus the reproducibility of the labyrinthopeptin A1 production phase, the recorded OTR values (Figure 5) were time‐shifted. The cultivation times for the OTR peaks were set to 3 days for the nonsupplemented cultures (see Figure 6A) and to 6 days for the salt‐supplemented cultures (see Figure 6B), indicated by a vertical dashed purple line.

For all nonsupplemented cultures, the time from the OTR peaks to the OTR drops to a plateau is approximately 2 days, indicating similar carbon source consumption dynamics (Figure 6A). Despite the large differences in their growth dynamics before reaching their respective OTR peaks, all salt‐supplemented cultures display a very similar OTR progression from 6 to 10 days (Figure 6B). As shown by Tesche et al. [32], the slightly increasing OTR plateaus between Days 7 and 10 of the cultivations from 2 to 2.5 mmol L^−1^ h^−1^ correlate with increased labyrinthopeptin A1 production of the salt‐supplemented cultures. Although the cultures show clear differences during their growth phase, the duration of the growth phase and oxygen consumption during the labyrinthopeptin A1 production phase are surprisingly similar.

The intOTR contains information on the overall metabolic activity of aerobic microorganisms in the time interval considered. As expected, intOTR of the salt‐enhanced cultures at the end of the cultivation time (Figure 6D) is higher compared to the nonsupplemented culture (Figure 6C), as the increased labyrinthopeptin A1 production is associated with increased oxygen consumption [32]. When comparing the intOTR courses of the nonsupplemented cultures, these cultures appear to follow the same progression overall. However, when comparing the intOTR at the time of the OTR peak after 3 days, the intOTR of the 2nd culture (139.2 ± 2.2 mmol L^−1^) is 35% higher than the intOTR of the 3rd culture (102.9 ± 2.6  mmol L^−1^, Figure 6C). Despite the differences in the initial growth behavior of the salt‐supplemented cultures, the intOTR values of the 2nd, 3rd, and 4th cultures differ by only less than 1% after 6 days, indicating very similar metabolic activity (Figure 6D). However, after 6 days, the two replicates of the 1st culture show an intOTR of 393.5 and 392.8 mmol L^−1^, respectively, which is 40% higher compared to the three other cultures. This is due to the higher oxygen consumption during the growth phase between Days 1 and 4 and may be related to a different morphology during the growth phase (Figure 6D). Unfortunately, several cultures showed excessive foaming after 10 days of cultivation, making further OTR monitoring impossible.

Although preculture heterogeneity mainly affects the lag‐phase duration of *A. namibiensis* cultures without salt‐supplementation, the addition of 50 mM (NH_4_)_2_SO_4_ degrades the reproducibility of the entire growth phase. Although salt‐supplementation increases labyrinthopeptin A1 production, the production phases of both conditions appear to be equally reliable. As shown, online monitoring techniques such as RAMOS offer the possibility to compare individual cultivation phases using the time‐shift method performed here.

## 4 Concluding Remarks

In this study, it is investigated, which rheometer setup is better suited to evaluate and compare the rheology of shear rate depending culture broths from shake flask cultivations of the filamentous bacterium *A. namibiensis*. As suggested by Bliatsiou et al. [19], both for the comparison of xanthan solutions as viscous model fluids and for the rheological characterization of *A. namibiensis* cultures, the PP rheometer setup provided more reproducible and reliable results than the VC tool. Based on these results, the PP rheometer setup is recommended for the investigation of the shear rate‐dependent viscosity of filamentous bacteria, when the pellet diameter is not more than one tenth of the PP gap. Supplementing the PP rheometer measurements with online monitoring of the specific power input provided further insights into the temporal evolution of the apparent viscosity of the culture broth.

The reproducibility of *A. namibiensis* cultures with and without addition of 50 mM (NH_4_)_2_SO_4_ was characterized based on biomass growth, substrate consumption, and labyrinthopeptin A1 production. It was found that the DOT and OTR values monitored online matched with the culture phases in terms of growth and production. This allowed the reproducibility of the culture to be assessed based on the observed differences over the course cultivation phases using the introduced time‐shift method. Although salt‐enhancement increases labyrinthopeptin A1 production, it decreases the reproducibility of the growth phase, as previously hypothesized by Tesche et al. [32]. Further studies are needed to evaluate the extent to which culture reproducibility can be performed for other filamentous organisms. To gain further insights, such studies may also include a deeper investigation of micro‐ and macromorphology, as performend by Tesche and Krull [9] for *A. namibiensis*, to link online data with morphogenesis.

This study highlights the importance of evaluating different rheometer systems for individual filamentous organisms and their morphological expression (e.g., size of agglomerates, pellets, mycelia, and clumps), before starting an in‐depth characterization or a scale transfer. In addition, online monitoring of DOT and OTR has been shown to provide valuable insights when evaluating culture reproducibility, as variation in media parameters, such as salt‐enhancement, can affect different phases of cultivation in ambiguous ways. As shown, the combination of OTR monitoring and rheological measurements enables the assessment of transitions in metabolic activity as well as morphological changes in the cultivation of *A. namibiensis*, allowing a better understanding of the complex filamentous culture behavior.

## Nomenclature

K
: flow consistency factor, Pa s*^m^*
m
: flow behavior index, –

### Greek Variables

$\dot{\gamma }$
: shear rate, $s^{-1}$
*η*_app_
: apparent viscosity, Pa s
τ
: shear stress, Pa
$\tau_{0}$
: yield stress, Pa

## Author Contributions

Conceptualization: Sebastian Tesche, Luc Fillaudeau, Jochen Büchs, and Rainer Krull. Formal analysis: René Hanke, Jonas Lohr, Leon Poduschnick, and Sebastian Tesche. Funding acquisition: Jochen Büchs and Rainer Krull. Investigation: René Hanke, Jonas Lohr, Leon Poduschnick, and Sebastian Tesche. Methodology: René Hanke, Jonas Lohr, and Sebastian Tesche. Resources: Jochen Büchs and Rainer Krull. Supervision: Jochen Büchs and Rainer Krull. Validation: Jochen Büchs and Rainer Krull. Visualization: René Hanke, Jonas Lohr, Leon Poduschnick, and Sebastian Tesche. Writing–original draft: René Hanke, Jonas Lohr, Leon Poduschnick, and Sebastian Tesche. Writing–review and editing: René Hanke, Jonas Lohr, Leon Poduschnick, Luc Fillaudeau, Jochen Büchs, and Rainer Krull. All authors have read and agreed to the published version of the manuscript.

## Conflicts of Interest

The authors declare no conflicts of interest.

## Supporting information



Supporting Information

## Data Availability

The data that support the findings of this study are available from the corresponding author upon reasonable request.
