# Supplementary material for: Rheology and Culture Reproducibility of Filamentous Microorganisms: Impact of Flow Behavior and Oxygen Transfer During Salt‐Enhanced Cultivation of the Actinomycete Actinomadura namibiensis
Source: Eng Life Sci. 2024 Nov 27;25(2):e202400078. doi: 10.1002/elsc.202400078 (PMC11842282; doi:10.1002/elsc.202400078)
Supplement: Supplementary file 1 — Supporting Information [file ELSC-25-e202400078-s001.pdf]

Supporting information for Research Article

Rheology and culture reproducibility of filamentous microorganisms: Impact of flow behavior and oxygen transfer during salt-enhanced cultivation of the actinomycete *Actinomadura namibiensis*

René Hanke[1*], Jonas Lohr[2,3*], Leon Poduschnick[1*], Sebastian Tesche[2.3], Luc Fillaudeau[4], Jochen Büchs[1], Rainer Krull[2,3]

[1]AVT.BioVT - Chair of Biochemical Engineering, Rheinisch-Westfälische Technische Hochschule (RWTH) Aachen University, Aachen, Germany

[2]Institute of Biochemical Engineering, Technische Universität Braunschweig, Braunschweig, Germany

[3]Center of Pharmaceutical Engineering (PVZ), Technische Universität Braunschweig, Braunschweig, Germany

[4]Toulouse Biotechnology Institute (TBI), Université de Toulouse, CNRS, INRAE, INSA, Toulouse, France

* René Hanke, Jonas Lohr and Leon Poduschnick have contributed equally to this study.

Correspondence: Prof. Rainer Krull (r.krull@tu-braunschweig.de). Institute of Biochemical Engineering, TU Braunschweig, Rebenring 56, 38106 Braunschweig, Germany.

Keywords: filamentous bacteria, online monitoring, Respiratory Activity Monitoring System (RAMOS), rheology, shake flasks

Determination of the broth viscosity based on online monitoring of the power input

The volumetric power input $(P/V)_{\varnothing}$ was calculated from the values of torque and shaking frequency with Eq. S1 (Büchs et al., 2000a).

$$(P/V)_{\varnothing} = 2 \cdot \pi \cdot n \cdot \frac{(M_L - M_S)}{V_L} \tag{S1}$$

with the shaking frequency n, the difference (M_L - M_S) between the torque M_L given by the torque measurements of the culture broth, and the reference torque M_S, which represents the mechanical friction of the system without cultivation broth. Thus, the difference between the two values determines the torque resulting from the movement of liquid inside the shake flasks, and the filling volume V_L.

The Modified Power number Ne' is calculated by Eq. S2

$$Ne' = \frac{P}{\rho \cdot n^3 \cdot d^4 \cdot V_L^{1/3}} \tag{S2}$$

with the power input P, the density of the culture broth ρ, the shaking frequency n, the shaking flask diameter d, and the filling volume V_L and by iteratively fitting Ne' calculated from the empirical relation given in Eq. S3 (Büchs et al., 2000b).

$$Ne' = \frac{70}{Re} + \frac{25}{Re^{0.6}} + \frac{1.5}{Re^{0.2}} \tag{S3}$$

Ne' and the apparent viscosity η_{app} of the culture broth η_{app} are related through the Reynolds number Re, given in Eq. S4.

$$Re = \frac{\rho \cdot d^2 \cdot n}{\eta_{\text{app}}} \tag{S4}$$

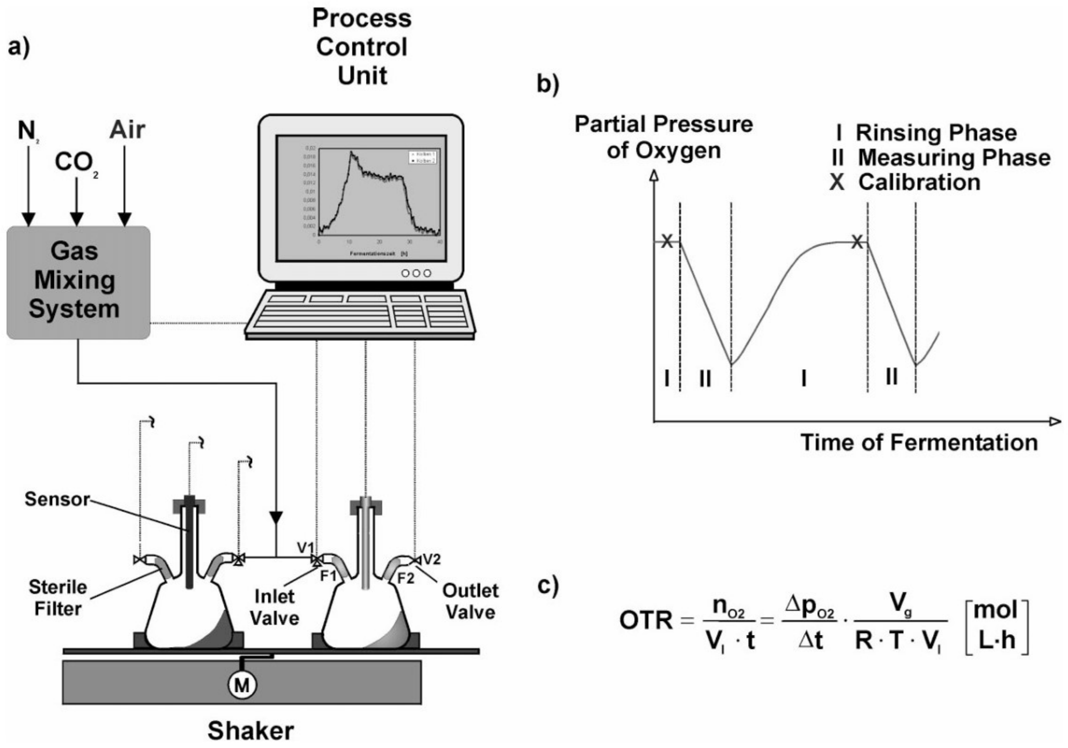

Figure S1: (a) Principle of the measuring device. (b) Partial pressure of oxygen during a measuring cycle. (c) Equation to determine the Oxygen Transfer Rate (OTR). Adapted from Anderlei and Büchs (2001) [27].

Table S1: Static yield stresses τ_0 with standard errors determined by fitting the Herschel-Bulkley equation (1) to the experimental data of shear rate tests in the range from 0.1 to 100 s^{-1} and coefficients of determination R^2 of the related fits. Adapted from Tesche (2020) [43].

**Cultivation time** [d]	τ_0 **(0 mM)** [mPa]	**R² (0 mM)** [-]	τ_0 **(50 mM)** [mPa]	**R² (50 mM)** [-]
0	2.1 ± 0.6	0.999	3.9 ± 0.8	0.999
1	14.7 ± 0.8	0.999	14.6 ± 3.6	0.985
2	54.5 ± 10.8	0.999	20.7 ± 10.9	0.991
3	75.6 ± 16.1	0.999	92.7 ± 75.6	0.996
4	163.1 ± 18.7	0.999	17.8 ± 47.2	0.996
5	225.5 ± 16.4	0.999	26.5 ± 57.8	0.987
6	51.7 ± 13.1	0.997	70.1 ± 37.9	0.999
7	69.9 ± 13.4	0.999	66.6 ± 52.5	0.998
8	0.0 ± 37.7	0.917	317.5 ± 46.7	0.999
9	0.0 ± 34.7	0.999	0.0 ± 36.8	0.997

The following paragraph is adapted from Tesche (2020) [43]:

"The static yield stress τ_0, which is required to initiate flow, is given in **Table S1**. The values of τ_0 vary between 0 and 317.5 mPa. The highest τ_0 values were determined on day 4 and 5 of cultivation in the non-supplemented culture, while the highest τ_0 values of the salt-enhanced culture were obtained on the cultivation day 6, 7 and 8. A similar transient maximum of τ_0 was observed in the cultivation of *T. reesei* (Marten et al. 1996, https://doi.org/10.1021/bp950066b). However, the data presented show high standard errors. The main reason for these errors is that there are many possible combinations of parameter values that give the same quality of fit. Nevertheless, the Herschel Bulkley model fitted the experimental data very well ($R^2 > 0.99$ in most cases). It should be noted that the accurate determination of the yield stress τ_0 is generally challenging and can be subject to many errors, e.g., due to wall slippage or damage to weak structures before the start of the shear test (Dinkgreve et al. 2016, https://doi.org/10.1016/j.jnnfm.2016.11.001; Kee 2021, https://doi.org/10.1063/5.0070209; TA Instruments, Rheological techniques for yield stress analysis. *TA Instruments Tech. Notes - RH025* 2000). Therefore, the determined values of τ_0 can only give an indication of the order of magnitude of the yield stress of the cultivation broths. The time-averaged yield stress was 66 and 58 mPa for the non-supplemented and the salt-enhanced culture, respectively. This is comparable to the yield stress of pulp-containing fruit juices (Diamante et al. 2015, https://doi.org/10.1080/10942912.2014.898653; Shamsudin et al. 2013, https://doi.org/10.1016/j.jfoodeng.2012.12.031)."

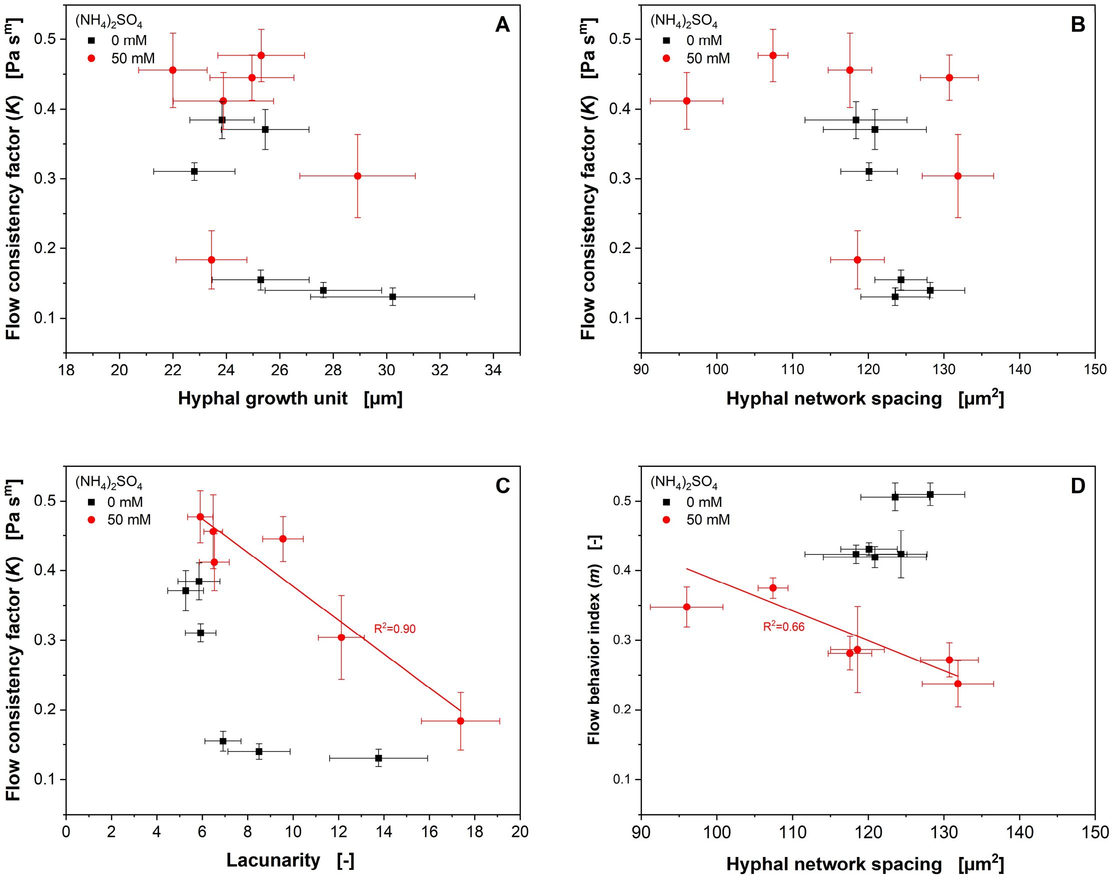

Figure S2: Morphological and rheological parameters of *A. namibiensis* cultures with and without 50 mM $(NH_4)_2SO_4$-supplementation of the culture medium: Plot of the flow consistency factor *K* against (**A**) hyphal growth unit, (**B**) hyphal network spacing, and (**C**) lacunarity. (**D**) Plot of the flow behavior index *m* against the hyphal network spacing. Error bars represent standard deviation of three biological replicates.

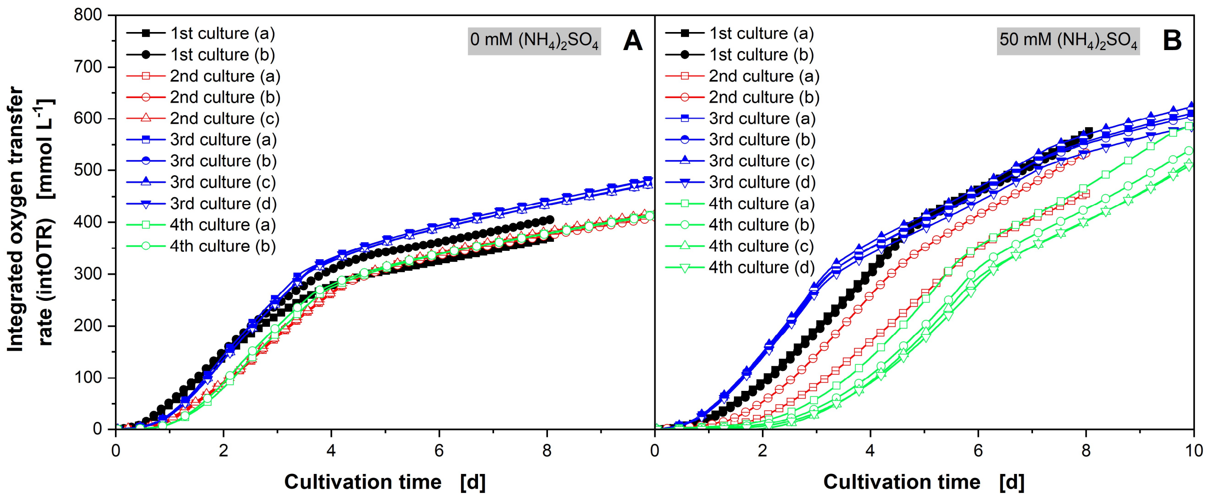

Figure S3: Integrated oxygen transfer rate (intOTR) of shake flask cultivations of *A. namibiensis* (**A**) without and (**B**) with supplementation of 50 mM $(NH_4)_2SO_4$, monitored using a RAMOS device. Four cultivations were performed with two to four replicates. The 2nd cultures (a) and (b) are already shown in Tesche et al. (2019). For clarity, only four data points per day are shown. The lines are drawn through all data points. The respective oxygen transfer rates are shown in **Figure 5**. V_F = 500 mL, V_L = 100 mL, T = 30 °C, n = 180 min^{-1}, d_0 = 50 mm.

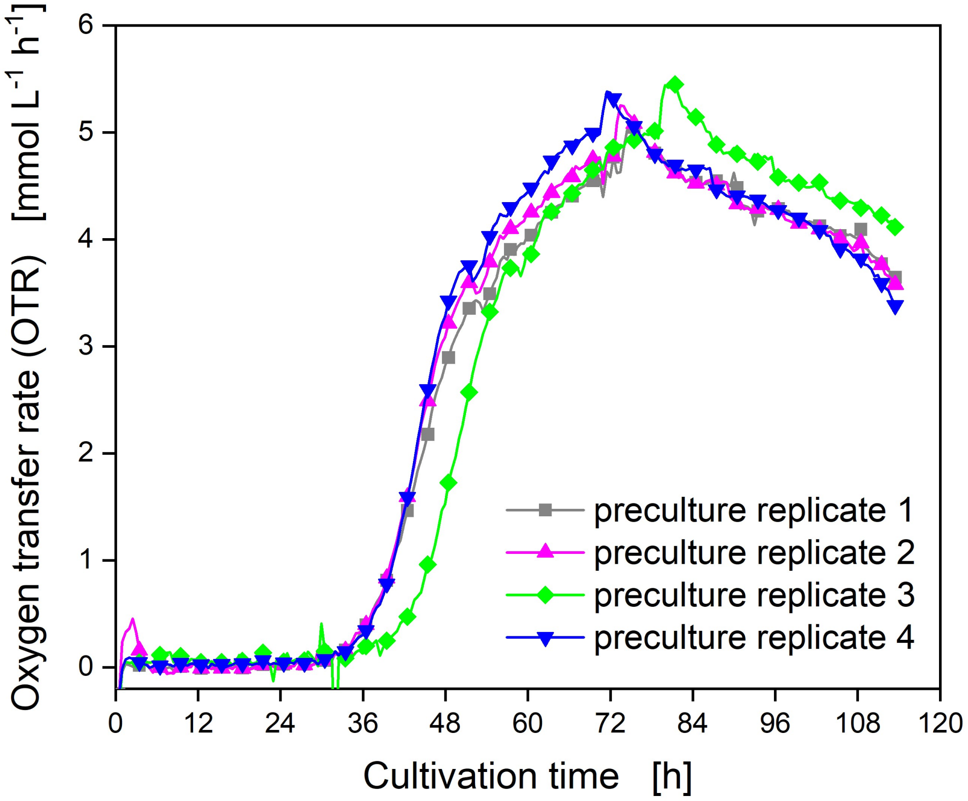

Figure S4: Shake flask preculture cultivations of *A. namibiensis* without salt supplementation, monitored using a RAMOS device. V_F = 250 mL, V_L = 70 mL, T = 30 °C, n = 180 min^{-1}, d_0 = 50 mm. Each preculture was inoculated with one thawed agar segment from the cryo culture. For a better visibility one every sixth data point is shown as symbol. The lines are drawn through all data points. Precultures were used for inoculation of main culture with and without salt-supplementation shown in **Figure 5**.

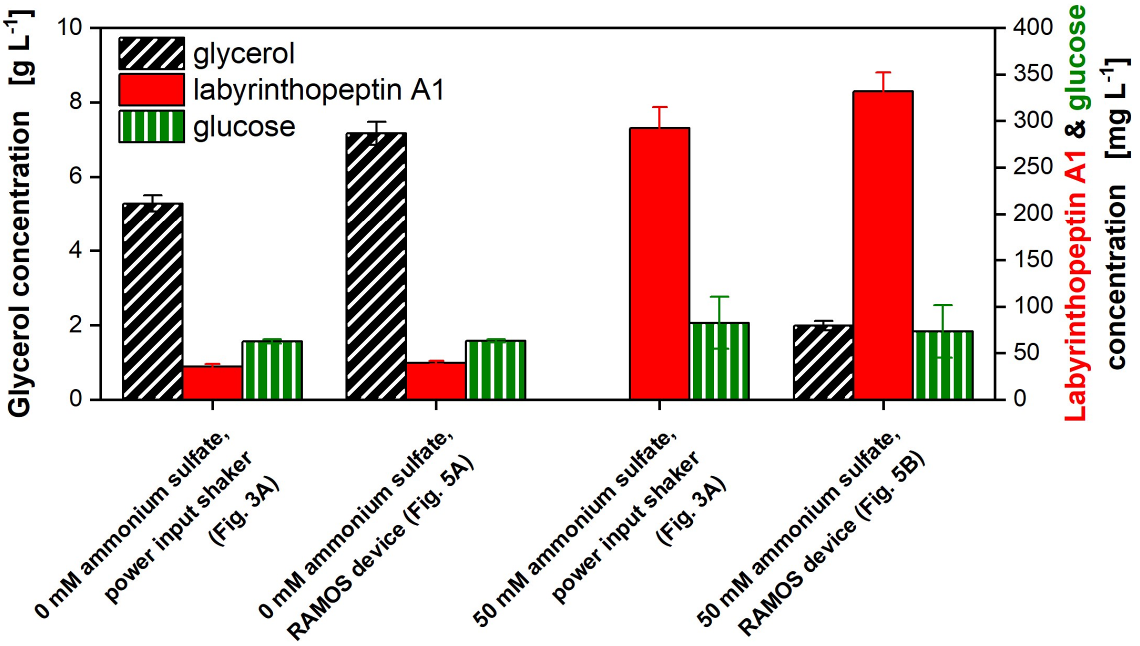

Figure S5: Substrate and product concentrations of *A. namibiensis* shake flask cultivations harvested after 10 days. Glucose, glycerol and labyrinthopeptin A1 concentrations are shown as average of three biological replicates. Standard deviation is indicated by error bars. The apparent viscosity η_{app} of cultures labeled "power input shaker" is given in **Figure 3A**. The oxygen transfer rate OTR of cultures labeled "RAMOS device" is given in "**Figure 5A, 3rd culture**" and "**Figure 5B, 3rd culture**", respectively. V_F = 500 mL, V_L = 100 mL, T = 30 °C, n = 180 min^{-1}, d_0 = 50 mm.
